# Aberrant methylation of *PSD* disturbs Rac1-mediated immune responses governing neutrophil chemotaxis and apoptosis in ulcerative colitis-associated carcinogenesis

**DOI:** 10.3892/ijo.2011.1301

**Published:** 2011-12-15

**Authors:** TAKAHARU KATO, KOICHI SUZUKI, SHINICHIRO OKADA, HIDENORI KAMIYAMA, TAKAFUMI MAEDA, MASAAKI SAITO, KEI KOIZUMI, YUICHIRO MIYAKI, FUMIO KONISHI

**Affiliations:** 1 Department of Surgery, Saitama Medical Center, Jichi Medical University, 1-847 Amanuma-cho, Omiya-ku, Saitama 330-8503; 2 First Department of Surgery, Hamamatsu University School of Medicine, 1-20-1 Handa-yama, Higashi-ku, Hamamatsu, Shizuoka 431-3192, Japan

**Keywords:** promoter methylation, *Pleckstrin and Sec7 domain-containing gene*, ulcerative colitis-associated colorectal carcinogenesis, apoptosis, Ras-related C3 botulinum toxin substrate 1, neutrophils, filamentous-actin

## Abstract

We previously reported that the *Pleckstrin and Sec7 domain-containing (PSD) gene* is preferentially methylated in patients with ulcerative colitis (UC) who developed colorectal cancer (CRC), and is implicated in UC-associated carcinogenesis through its inhibition of apoptosis. This study aimed to determine the potential effect of *PSD* methylation on its downstream molecule, Ras-related C3 botulinum toxin substrate 1 (Rac1), which governs neutrophil chemotaxis and apoptosis signaling. *PSD* was knocked down in a normal human fibroblast cell line (HNDF) and a neutrophil-like cell line (HL-60). Both NHDF and HL-60 cells exhibited numerous filamentous-actin (F-actin) rich membrane extensions, resulting in the activation of Rac1; this activation was hampered by *PSD* silencing. Lipopolysaccharide, a reactive oxygen species (ROS) inducer, stimulated NHDF cells to release ROS and activated caspase-3/7 in the presence of neutrophils, which was inhibited by *PSD* knockdown. Migration assays demonstrated that chemotaxis of HL-60 cells was affected by *PSD* silencing in NHDF cells. Tissue sections from 6 UC patients with CRC and 15 UC patients without CRC were examined. To verify Rac1-mediated chemotaxis in tissue sections, we evaluated the grade of neutrophil infiltration by histological assessment and assessed F-actin and PSD expression by immunohistochemistry. Neutrophil infiltration, F-actin and PSD expression were significantly decreased in specimens from UC patients with *PSD* methylation compared with those without. Decreased levels of F-actin expression were observed in colorectal mucosa, as well as in infiltrating cells with *PSD* methylation. PSD expression was preferentially inhibited in colorectal mucosa by *PSD* methylation, whereas PSD expression was rarely observed in infiltrating cells, regardless of *PSD* methylation status. These data indicate that aberrant methylation of *PSD* occurs in UC-associated colorectal mucosa, enabling circumvention of Rac1-mediated immune responses governing neutrophil chemotaxis and apoptosis, and thus plays a pivotal role in the mechanisms underlying UC-associated carcinogenesis.

## Introduction

Ulcerative colitis (UC) is a chronic inflammatory bowel disease (IBD), the etiology of which is not completely understood. Patients with UC face an increased risk of developing colorectal cancer (CRC) (1,2). Recent advances in IBD research have provided genetic insights into its pathogenesis. The innate and adaptive immune system (3–5), autophagy pathway (6–8), and epithelial barrier (9,10) participate in fighting pathogens involved in IBD. Interaction between host and pathogens leads to persistent or severe inflammation, whereas insufficient interaction may result in failure to prevent cancer development.

A genome-wide association study previously identified genetic variants in *Disks large homolog 5* (*DLG5*) gene associated with IBD (10). *DLG5* regulates cell shape, polarity (11) and cell-cell contact (12), disruption of which interferes with the epithelial barrier function in the colon. In our previous study, genome-wide analysis of methylation alterations using a methylation-sensitive representational difference analysis identified the *Pleckstrin and Sec7 domain-containing (PSD)* gene (13), which has similar roles to *DLG5*, such as coordination of cell shape and polarity *PSD* was more frequently methylated in both UC-associated colorectal cancer tissues (71.4%) and matched normal epithelia (57.1%) than in non-neoplastic UC epithelia (27.3%) and sporadic colorectal cancer tissue (18.8%). In addition, silencing of *PSD* inhibited apoptosis in a fibroblast cell line and in tissue specimens from UC patients harboring *PSD* methylation. These findings led us to address the potential roles of *PSD* methylation in the mechanisms underlying UC-associated carcinogenesis.

*PSD* regulates Ras-related C3 botulinum toxin substrate 1 (Rac1), a Rho GTPase. Rac1 is implicated in the regulation of neutrophil functions in response to inflammatory signals, including actin remodeling, chemotaxis and production of NADPH oxidase. Rac1 is reported to induce apoptosis in response to UV light (14) and other damaging agents such as Fas (15) and TNF-α (16). These findings indirectly support our data that previously reported *PSD* silencing and methylation inhibited apoptosis *in vitro* and in tissue specimens, respectively. In this study, we elucidated the effect of *PSD* methylation on Rac1, which governs neutrophil chemotaxis and apoptosis signaling, in a normal human fibroblast cell line (HNDF) and a human promyelocytic leukemia cell line (HL-60), which look and behave like neutrophils (17–19), and in tissue sections from UC patients with and without CRC.

## Materials and methods

### Patients and tissues

Six samples of UC-associated colorectal cancer tissue (UCT) with matched normal epithelial tissue (UCN), and 15 samples of non-neoplastic UC epithelial tissue (UCI) were obtained from patients who had undergone surgery at Jichi Medical University Saitama Medical Center and Jichi Medical University Hospital between November 2000 and September 2006. Matched normal epithelia were taken from lesions harboring colitis adjacent to tumors. This study was approved by the Ethics Committee of Jichi Medical University, and informed consent was obtained from each participant.

### Cell lines

A human skin fibroblast cell line, NHDF, was obtained from Kurabo (Osaka, Japan) and was maintained in medium 106S supplemented with low-serum growth supplement. A human promyelocytic leukemia cell line, HL-60, which orient their polarity in response to a gradient and migrate towards the stimulus (17–19), were obtained from the Japanese Collection of Research Bioresources (JCRB, Osaka, Japan). HL-60 cells were maintained in RPMI-1640 medium supplemented with 10% heat-inactivated fetal bovine serum.

### Quantitative reverse transcription-PCR

Tissue specimens were immediately added to RNA later (Ambion, Austin, TX, USA) and stored at −80°C until DNA or RNA extraction. Total RNA was immediately treated with DNase I (Invitrogen, Carisbad, CA, USA) and reverse-transcribed using a Superscript II reverse trans-criptase kit (Invitrogen) to prepare first-strand cDNA. The primer sequences for *PSD* were 5′-CCATAGACGAGGAGGAGCTG-3′ (forward) and 5′-TCTTCCTGCAGTCAGGGTCT-3′ (reverse). Thermal cycling conditions were 42°C for 60 min (cDNA synthesis), 95°C for 10 sec (hot start), and then 40 cycles of 95°C for 5 sec, 58°C for 10 sec, and 72°C for 30 sec. The expression level of *PSD* was determined using the fluorescence intensity measurements from the ABI 7900HT Real-Time PCR System Data Analysis Software. A GAPDH fragment was amplified as an internal control.

### Knockdown of PSD in HNDF and HL-60 cells

PSD-specific siRNA (siPSD) was purchased from Invitrogen. RNA oligonucleotides were resuspended in 10 μM Tris-HCl, pH 8.0, 20 mM NaCl, and 1 mM EDTA to make a 20 μM siRNA solution. The final siRNA concentration was 30 nM in Opti-MEM I without serum. HNDF and HL-60 cells were cultured in dishes at 30–50% confluency without antibiotics, and transfection was performed with Lipofectamine 2000 (Invitrogen) according to the manufacturer’s instructions. The Block-iT™ Fluorescent Oligo (Invitrogen), a fluorescently labeled double-stranded RNA duplex with the same length, charge, and configuration, was used for the assessment of transfection efficiency, and Scrambled Stealth™ RNA molecule was used as the control siRNA (siControl). Cells were incubated for 48 h after transfection at 37°C in a CO_2_ incubator, and used for subsequent experiments.

### Actin cytoskeleton analysis

si*PSD*-treated and siControl-treated cells were stained with fluorescent rhodamine-labeled phalloidin using a F-actin Visualization BiochemKit™ (Cytoskelton, Denver, USA) according to the manufacturer’s instructions. Cells were stimulated with EGF (10 ng/ml for 1, 2 and 30 min) or calpeptin (0.1 mg/ml for 30 min) after treatment with si*PSD* or siControl for 48 h and subsequently stained with rhodamine-labeled phalloidin. Hoechst 33342 was used to observe nuclear morphology. Signals were observed by a fluorescence microscope (Fluoview FV500; Olympus) with excitation at 535 nm and emission at 585 nm for the detection of rhodamine, and with excitation at 365 nm and emission at 480 nm for the detection of Hoechst 33342. EGF was purchased from Wako (Tokyo, Japan) and Hoechst 33342 was included in the Total ROS/Superoxide Detection kit™ (Enzo, PA, USA).

### Measurement of Rac1 activity

The activity of Rac1 was measured by G-LISA™ Rac Activation Assay Biochem kit™ (Cytoskelton) according to the manufacturer’s instructions. Cells were stimulated with EGF (10 ng/ml for 1, 2 and 30 min) or calpeptin (0.1 mg/ml for 30 min) after treatment with si*PSD* or siControl for 48 h and then collected in 100 μl of ice-cold lysis buffer provided by G-LISA. Lysates were centrifuged to remove cellular debris. From each supernatant, 10 μl was removed to measure protein content using the Precision Red™ Advanced Protein Assay Reagent included in the G-LISA Rac Activation Assay Biochem kit™, and 50 μl was used for the G-LISA Rac activation assay, followed by dilution with 50 μl of cold binding buffer. Lysate (50 μl) was added into respective wells in the Rac1-binding plate for duplicate assays, and then the plate was put on a cold orbital microplate shaker at 4°C for exactly 30 min. Next, the wells were washed twice with wash buffer. Antigen (200 μl) presenting buffer was immediately added into each well and the plate was incubated at room temperature for 2 min. Wells were then washed three times with wash buffer. Next, 50 μl of diluted anti-Rac1 primary antibody was added to each well and the plate was placed on an orbital microplate shaker at room temperature for 45 min. Next, 50 μl of diluted anti-Rac1 secondary antibody was added to each well and the plate was incubated for 45 min, followed by incubation with 50 μl of HRP detection reagent for 20 min. Immediately after addition of 50 μl of HRP Stop Buffer, the absorbance was read using a microplate spectrophotometer.

### Detection of reactive oxygen species and active caspase-3/7

Reactive oxygen species (ROS) and active caspase-3/7 were detected by Total ROS/Superoxide Detection kit (Enzo) and CaspaTag™ Caspase-3/7 Assay In Situ Assay Kit (Chemicon), respectively, according to the manufacturer’s instructions. After treatment with si*PSD* or siControl for 48 h, NHDF cells were exposed to lipopolysaccharide (20 ng/ml; Wako) and were co-cultured with HL-60 cells for an additional 48-h period. Then, HL-60 cells were removed and NHDF cells were subjected to the ROS detection assay or the caspase-3/7 activity assay. Fluorescence signals were subsequently detected by a fluorescence microscope (Fluoview FV500; Olympus), with excitation at 490 nm and emission at 525 nm for the detection of ROS, and with excitation at 550 nm and emission at 580 nm for the detection of caspase-3/7, respectively. Average number of cells inducing ROS or expressing caspase-3/7 in three random fields was calculated.

### Migration assay

Neutrophil chemotaxis in response to inflammation *in vitro* was assessed by migration assays using a BD Falcon companion plate and cell culture insert (Becton-Dickinson) with a 3-μm pore size. After NHDF cells were treated with si*PSD* or siControl for 48 h in the bottom wells (24-well companion plate, Becton-Dickinson), they were exposed to LPS and cultured with HL-60 cells seeded in culture inserts for an additional 48 h. Then, non-migrating cells were removed from the insert membranes by cotton swabs. The membrane was mounted onto a slide glass and the nuclei of migrated cells were then stained with Hoechst 33342. The number of migrated cells was counted in three random fields using an inverted microscope.

### Histological grades of neutrophil infiltration in tissue sections

Histological grades of neutrophil infiltration were determined using a scoring system as previously described (20): 0, normal (no inflammatory cells); 1, mild active; 2, moderate active (with cryptitis). The average grading of three regions of the colorectum (rectum, descending colon and ascending colon) were calculated where samples were available.

### Detection of filamentous actin and PSD in tissue specimens

Detection of filamentous actin (F-actin) and PSD in paraffin-embedded tissue sections was performed by immunohistochemical analysis using the I-View DAB Universal kit (Roche, Rotkreuz, Switzerland). Sections were de-waxed in xylene and rehydrated with distilled water before analysis, then treated with a heat-induced epitope retrieval technique using an EDTA buffer at pH 9.0, and blocked for endogenous peroxidase activity before addition of the primary antibody. NH3 and ab5962 (Abcam, Tokyo, Japan) were used as primary antibodies for F-actin and *PSD*, respectively. Incubation with primary antibody was performed overnight at 37°C. Cells displaying slight staining of the cytoplasm were determined to be positive. A grading system was applied to the assessment of accumulation of F-actin and PSD expression in tissue sections. The F-actin and PSD index were calculated based on percentage of staining cell, with 0, +1, and +2 when <−5% of cells, 5–20% cells, and >20% cells demonstrated cytoplasm reactivity, respectively. The average grading of three regions of the colorectum (rectum, descending colon and ascending colon) was calculated when samples were available.

### Statistical analysis

Values are shown as mean ± SE. Statistical differences between variables were determined by use of an unpaired t-test or an analysis of variance, as appropriate. Simple regression coefficient analysis was used to examine associations between two categorical variables. Values of P<0.05 were considered significant.

## Results

### Clinicopathological features

The clinicopathological features of patients recruited for this study are shown in Tables I and II. There was no significant difference in the average age. The disease duration of UCI patients (8.0±5.0 years) was significantly shorter than that of the UCT patients (14.8±7.0 years, P<0.05). Aberrant methylation of *PSD* was observed in 4 of 6 UCT patients (71.4%), 4 of 6 UCN patients (57.1%) and 5 of 15 UCI patients (27.3%), respectively, as previously reported.

### Knockdown of PSD inhibited membrane ruffling and reduced Rac1 activity in NHDF and HL-60 cells

**Figure 1:**
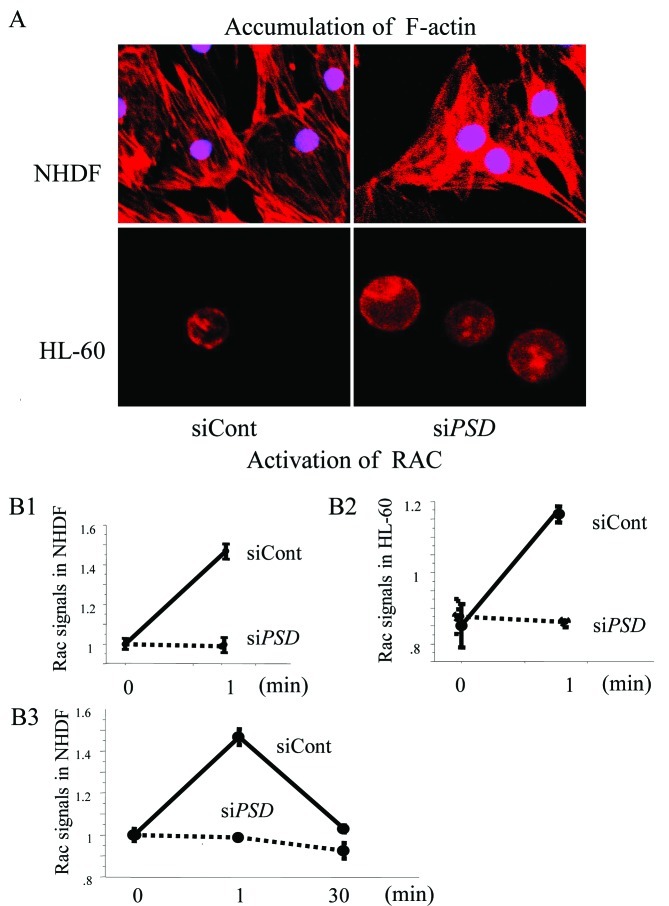
(A) Rhodamine-labeled actin (red) polymerizes preferentially at the edge of lamellipods in siControl-treated NHDF and HL-60 cells (left) after stimulation with EGF, whereas rhodamine-labeled actin remained inside the plasma membrane in si*PSD*-treated NHDF and HL-60 cells (right). Nuclei were stained with Hoechst 33342 (pink). (B) Rac1 signaling before and after stimulation with EGF in siControl-treated and si*PSD*-treated NHDF cells (B1) and in siControl-treated and si*PSD*-treated HL-60 cells (B2). (B3) Rac1 signaling declined to basal levels by 30 min after stimulation.

To determine the inhibitory effect of *PSD* silencing on the activation of Rac1, NHDF and HL-60 cells were treated with *PSD*-specific siRNA (si*PSD*) or siControl. Transfection efficiency was 89.1% in NHDF cells and 73.0% in HL-60 cells, which reduced the mRNA levels of PSD by 90.1% in siPSD-transfected NHDF cells and 61.3% in siPSD-transfected HL-60 cells, respectively. *PSD* promotes numerous F-actin-rich membrane extensions (21), which leads to the activation of Rac1. The accumulation of F-actin by stimulation with EGF was visualized by staining with fluorescent rhodamine-labeled phalloidin. EGF-stimulated membrane ruffling was observed in both siControl-treated NHDF and HL-60 cells, whereas no morphological changes of the membrane were found in either si*PSD* treated-NHDF or HL-60 cells (Fig. 1A). Likewise, Rac1 activity was increased in both siControl-treated NHDF and HL-60 cells after stimulation with EGF (Fig. 1B1 and B2), whereas activation was hindered in either si*PSD*-treated NHDF or HL-60 cells. Stimulation with calpeptin for 30 min also resulted in increased levels of Rac1 in both siControl-treated NHDF and HL-60 cells (data not shown). Rac1 activity declined to basal levels within half an hour after stimulation with EGF (Fig. 1B3), which was consistent with the results reported by Kurokawa *et al* (22).

### Knockdown of PSD in NHDF cells prevented induction of ROS and ROS-induced caspase-3/7 activity in the presence of HL-60 cells

**Figure 2:**
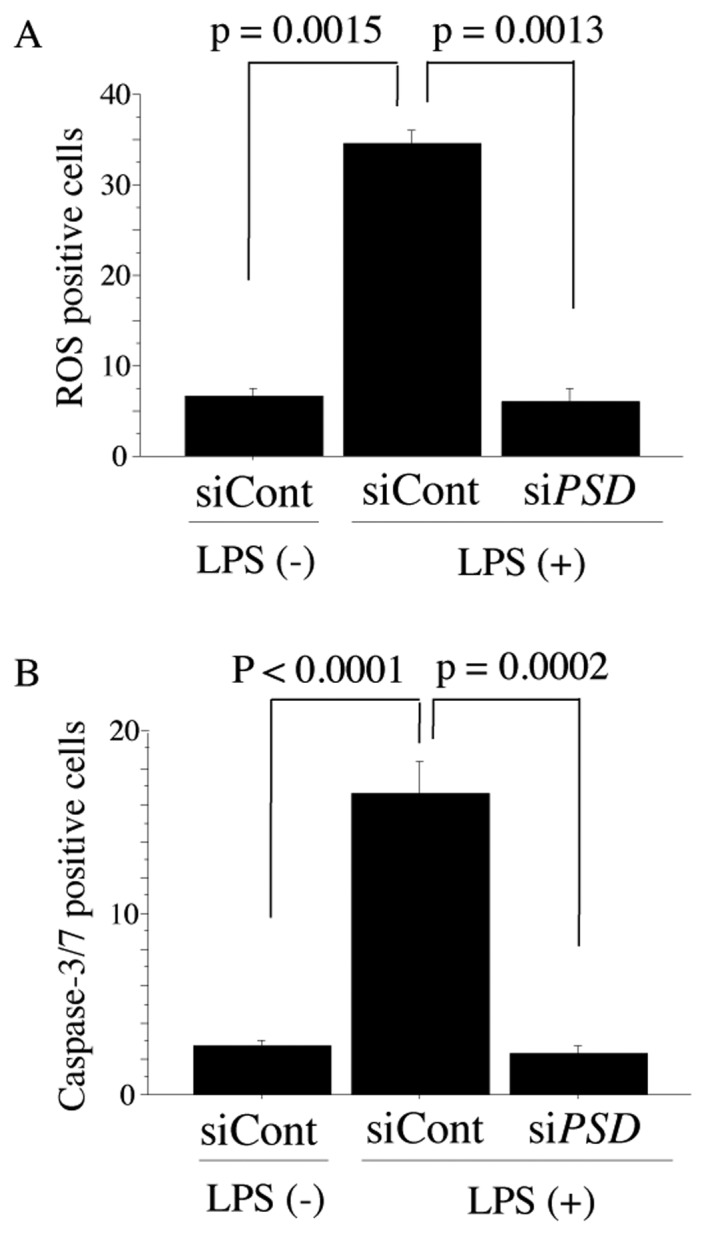
Average number of NHDF cells inducing ROS (A) and expressing active caspase-3/7 (B). After treatment with si*PSD* or siControl for 48 h, NHDF cells were exposed to lipopolysaccharide (LPS; 20 ng/ml) for 48 h in the presence of HL-60 cells. Then, HL-60 cells were removed and NHDF cells were subjected to ROS detection assays or caspase-3/7 activity assays.

**Figure 3:**
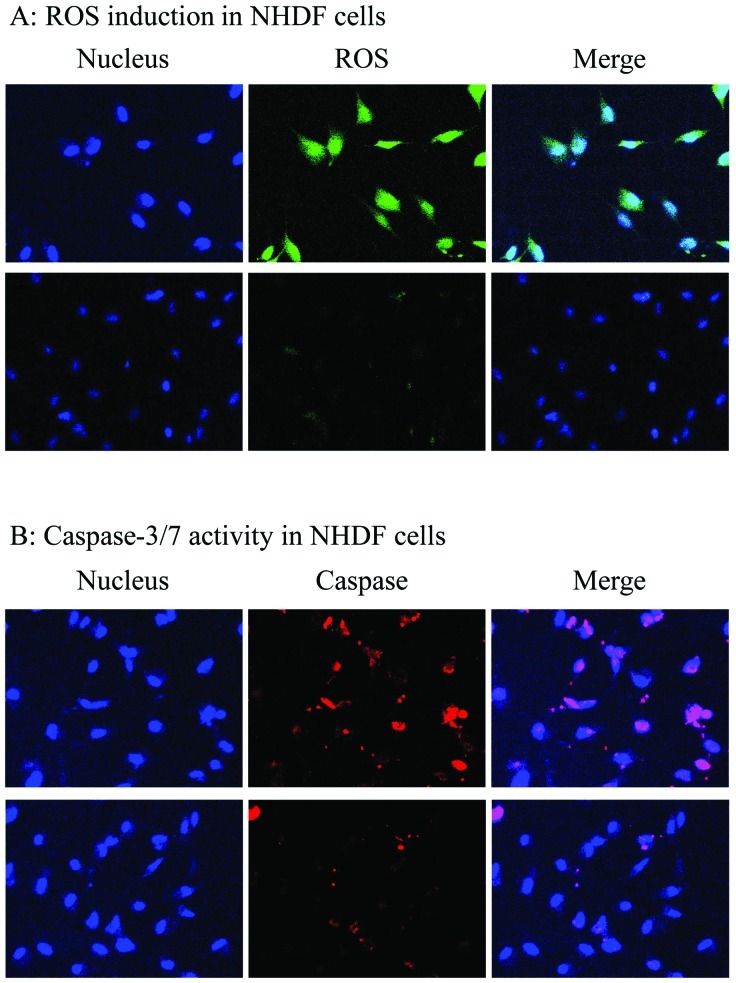
(A) Cells inducing reactive oxygen species (ROS) in siControl-treated (top panel) and si*PSD*-treated NHDF cells (bottom panel). Nuclear morphology of cells stained with Hoechst 33342 (blue, left), cells inducing ROS stained with Total ROS/Superoxide Detection kit Reagent (green, middle), and merged staining with both reagents (white green, bottom). (B) Cells expressing active caspase-3/7 in siControl-treated (top panel) and si*PSD*-treated NHDF cells (bottom panel). Nuclear morphology of cells stained with Hoechst 33342 (blue, left), cells expressing active caspase-3/7 stained with CaspaTag Reagent (red, middle), and merged staining with both reagents (pink, right).

Our previous study showed that NHDF cells were stimulated to release reactive oxygen species (ROS) by pyocyanin-harboring redox reactions, while lipopolysaccharide (LPS), which mediates the activation of NADPH oxidation in neutrophils, did not stimulate NHDF cells (13). In the present study, we attempted to elucidate whether LPS stimulates NHDF cells to release ROS when co-cultured with HL-60 cells, and if ROS induction is inhibited by *PSD* silencing in NHDF cells because they co-exist and interact in the body. LPS stimulated siControl-treated NHDF cells to release ROS in the presence of HL-60 cells, whereas LPS did not stimulate si*PSD*-treated NHDF cells to release ROS (Fig. 2A; 16.67±3.06 in siControl vs. 2.67±10.58 in si*PSD*, P=0.0013). Representative data for siControl-treated and si*PSD*-treated NHDF cells are shown in Fig. 3A. Next, we accessed ROS-mediated caspase-3/7 activation. Activation of caspase-3/7 was observed in siControl-treated NHDF cells, but was not noted in si*PSD*-treated NHDF cells (Fig. 2B; 34.67±2.51 in siControl vs. 6.00±2.65 in si*PSD*, P=0.0002). Representative caspase-3/7 positive cells detected by the CaspaTag Caspase-3/7 Assay In Situ Assay Kit (Chemicon) and cell nucleus stained with Hoechst 33342 in NHDF cells treated with si*PSD*or siControl are shown in Fig. 3B.

### Chemotaxis of HL-60 cells was disturbed by PSD silencing in NHDF cells

**Figure 4:**
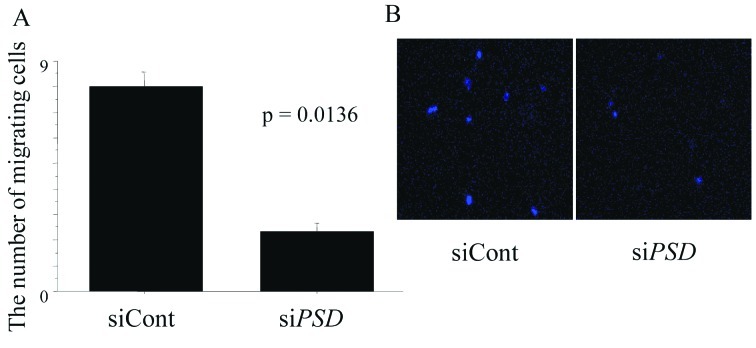
(A) Average number of NHDF cells migrating through membrane as detected by migration assay. After NHDF cells were treated with si*PSD* or siControl for 48 h in the bottom wells (24-well companion plate), cells were exposed to LPS and cultured with HL-60 cells in culture inserts for additional 48 h. The number of migrated HL-60 cells was counted in three random fields using an inverted microscope. (B) Microscopic images of migrated NHDF cells.

NHDF cells exhibited induction of LPS-mediated ROS and activation of caspase-3/7 when cultured with HL-60 cells, which was not observed in NHDF cells cultured alone or siPSD-treated NHDF cells co-cultured with HL-60 cells, suggesting that the interaction between activated NHDF cells and HL-60 cells was involved in this process. To investigate whether LPS-mediated *PSD* activation in NHDF cells affects the chemotaxis of HL-60 cells, migration assays were performed. Fig. 4 shows microscopy images of cells in a migration assay for each experimental setting. The number of migrated HL-60 cells co-cultured with siControl-treated NHDF cells was 4.6 times greater than HL-60 cells co-cultured with si*PSD*-treated HL-60 cells (Fig. 4A; 2.667±0.882 in siControl vs. 12.333±1.453 in si*PSD*, P=0.0047). Microscopic images of migrated NHDF cells are shown in Fig. 4B.

### The level of neutrophil infiltration was significantly decreased in specimens from patients with PSD methylation

**Figure 5:**
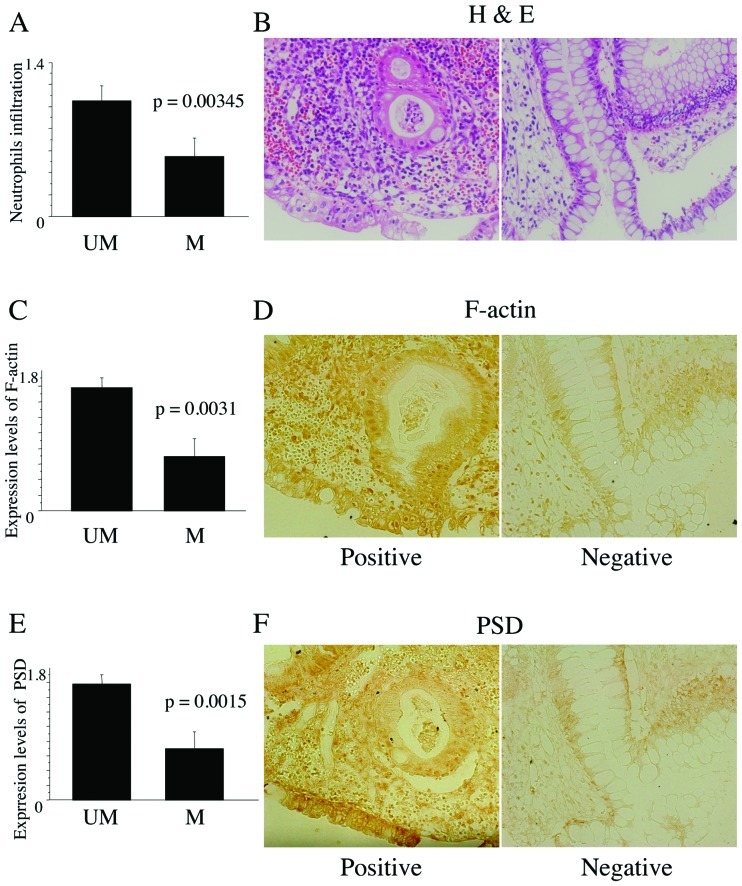
(A) Histological grades of neutrophil infiltration in tissue specimens from UC patients with (M) and without *PSD* methylation (UM). For evaluation, histological grades of inflammation were determined using a scoring system based on epithelial neutrophils as previously described (19): 0, normal (no inflammatory cells); 1, mild active; 2, moderate active (with cryptitis). The average grading from three regions of the colorectum was calculated. (B) Representative neutrophil infiltration in tissue specimens from UC patients without (left) and with *PSD* methylation (right). (C) The F-actin index in tissue specimens from UC patients with (M) and without *PSD* methylation (UM). For evaluation, three grades were determined as 0, +1, and +2 when <5% of cells, 5–20% cells, and >20% cells demonstrated cytoplasm reactivity, respectively. The average grading from three regions of the colorectum was calculated. (D) Representative positive (right) and negative cells for F-actin (left) in tissue specimens from UC patients without and with *PSD* methylation, respectively. (E) The *PSD* index in tissue specimens from UC patients with (M) and without *PSD* methylation (UM). For evaluation, three grades were determined as 0, +1, and +2 when <5% of cells, 5–20% cells, and >20% cells demonstrated cytoplasm reactivity, respectively. The average grading from three regions of the colorectum was calculated. (F) Representative positive (right) and negative cells for *PSD* in tissue specimens from UC patients without and with *PSD* methylation, respectively.

To verify if *PSD* methylation affected neutrophil chemotaxis in tissue specimens, the infiltration of neutrophils was evaluated by histological assessment. Neutrophil infiltration was significantly decreased in specimens from patients with *PSD* methylation than in those without (Fig. 5A; 0.51±0.55 with vs. 1.01±0.55 without, P=0.0166). Regardless of cancer status, the level of neutrophil infiltration was significantly decreased in both UCT and UCN when compared with UCI (0.29±0.49 in UCT vs. 1.1±0.55 in UCI, P=0.0018; 0.39±0.32 in UCN vs. 1.1±0.55 in UCI, P=0.0034). Representative data from tissue specimens from UC patients without and with *PSD* methylation are shown in Fig. 5B.

### Accumulation of F-actin was decreased in tissue specimens from UC patients with PSD methylation

To determine the distribution of PSD promoting accumulation of F-actin in tissue specimens, immunohistochemistry was performed. F-actin levels were significantly decreased in specimens from UC patients with *PSD* methylation compared to those without (Fig. 5C; 0.69±0.86 with vs. 1.57±0.51 without, P=0.0031), suggesting that accumulation of F-actin was inhibited by *PSD* methylation. This change was seen not only in colorectal mucosa but also in infiltrating cells. Representative F-actin-positive and -negative cells in tissue specimens are shown in Fig. 5D.

### PSD expression was decreased in tissue specimens from UC patients with PSD methylation, and was preferentially suppressed in epithelial cells rather than neutrophils

To clarify the distribution of PSD expression in tissue specimens, immunohistochemical analysis was performed. The expression level of *PSD* was significantly decreased in specimens from UC patients with *PSD* methylation compared with those without (Fig. 5E; 0.727±0.141 with vs. 1.462±0.144 without, P=0.0015), and was significantly correlated with the methylation status of *PSD*. In addition, immunohistochemical analysis revealed that PSD expression was inhibited in colorectal mucosa with methylated *PSD*, whereas PSD was rarely expressed in infiltrating cells regardless of *PSD* methylation status. These results indicated that aberrant methylation of *PSD* in UC-associated colorectal mucosa circumvented neutrophil chemotaxis, which resulted in the disturbance of neutrophil-regulated apoptosis. Representative *PSD*-positive and -negative cells are shown in Fig. 5F.

## Discussion

The present study demonstrated the crucial role of *PSD* silencing in NHDF cells through its inhibitory effect on Rac1 activation, which disturbed membrane ruffling and chemotaxis of HL-60 cells and consequently hampered the apoptotic machinery. In tissue specimens from UC patients, aberrant methylation of *PSD* interfered with actin rearrangement, neutrophil infiltration and apoptosis. Taken together, these data indicate that aberrant methylation of *PSD* in UC-associated colorectal mucosa circumvents Rac1-mediated immune responses, neutrophil chemotaxis and the apoptotic machinery, and thus likely plays a pivotal role in the mechanisms underlying UC-associated carcinogenesis.

The small GTPases have been implicated in diverse biological functions such as cytoskeleton rearrangement, cell growth, transformation, cell motility, migration, metastasis, and response to stress. One of these GTPases, Rac1, is reported to play a crucial role in inducing apoptosis in response to several stimuli such as UV light (14), and other damaging agents such as Fas (15) and TNF-α (16). These findings strongly support our data showing that Rac1-mediated apoptosis was inhibited by *PSD* silencing in si*PSD*-treated cells and was decreased in tissue specimens from UC patients harboring *PSD* methylation. Some reports, however, have presented conflicting results (23–25), raising the possibility that Rac1 has a complex role, and is involved in both inhibition and stimulation of apoptosis. This dual role in cell proliferation and apoptosis has been observed for other Rho proteins such as oncogenic *vav* (16) and R-*ras* (26,27). Esteve *et al*, explain that a dual role of Rho proteins in the regulation of cell apoptosis comes from the evidence that Rho proteins induce activation of the pathway leading to the JNK/SPAK cascade in several cell systems as well as nuclear factor κB (16). The fate of cells as determined by these molecules still remains to be explored.

Rho GTPases coordinate many cellular responses, often by regulating formation of different actin assemblies. *PSD* promotes numerous F-actin-rich membrane extensions (21), which leads to the activation of Rac1. Our results revealed that the accumulation of F-actin was significantly decreased in si*PSD*-treated NHDF and HL-60 cells, and in tissue specimens from UC patients harboring methylated *PSD*, which indicated that *PSD* methylation abolished F-actin-induced membrane extensions and consequently inactivated Rac1. Our findings concur with the report by Srinivasan *et al*, that a dominant-negative Rac1 mutant inhibits chemoattachment-stimulated accumulation of F-actin and polarization (28). A significant correlation between a decreased level of apoptosis and the accumulation of F-actin in specimens from UC patients with *PSD* methylation indicated that *PSD* methylation inactivated Rac1 and consequently abrogated Rac1-mediated apoptosis.

In considering alternative participants in the immune system underlying inflammatory bowel disease, neutrophils should be taken into consideration. Neutrophils are recruited from the circulation to take part in the defense against infectious agents but they may also cause tissue destruction in the host by secretion of toxic granule proteins and ROS (29). In this machinery, Rac1 also plays a crucial role in neutrophil migration and oxygen radical generation through NADPH oxidase. Glogauer *et al* demonstrated that infiltration of neutrophils was decreased in Rac1-null mice compared to wild-type mice (30). Delayed accumulation of neutrophils was also observed in Rac1-null mice. In addition, Srinivasan *et al* (28) demonstrated that a dominant-negative Rac1 mutant inhibited chemoattachment-stimulation. In accordance with these observations, our results revealed that the accumulation of neutrophils was significantly decreased in specimens from patients with *PSD* methylation. The inactivation of Rac1 resulting from *PSD* methylation may lead to delayed recruitment of neutrophils or disturbance of neutrophil chemotaxis. In the present study, we demonstrated that *PSD*-promoted accumulation of F-actin was decreased in colorectal mucosa, as well as infiltrating cells from ulcerative colitis patients with *PSD* methylation. PSD expression was preferentially inhibited in colorectal mucosa by *PSD* methylation, whereas PSD expression was rarely observed in infiltrating cells regardless of *PSD* methylation status. Considering the short half-life of neutrophils, it is unlikely that they would be methylated, indicating that colorectal mucosa could be targeted for aberrant methylation under circumstances of chronic inflammation such as ulcerative colitis, leading to neutrophil dysfunction.

Oxidative stress occurs in connection with inflammatory bowel disease. Neutrophils participate in this mechanism to release ROS, leading to protein damage, lipid peroxidation, and DNA damage. This results in genetic and epigenetic alterations, which pave the way for increasing grades of dysplasia and carcinoma. Furthermore, ROS have important functions in intracellular signaling, partly with anti-carcinogenic effects such as triggering apoptosis (31). Inadequate interaction between neutrophils and colorectal mucosa by *PSD* methylation could result in the disruption of the immune system in inflammatory bowel disease, which would be implicated in the mechanisms underlying UC-associated carcinogenesis. However, apoptotic pathways mediated by *PSD* signaling still remain to be considered as direct effectors on UC-associated colorectal mucosa to induce apoptosis.

Although the number of samples included in this study was limited and further investigations are required to draw definitive conclusions, the present study demonstrates that the inter-action between colorectal mucosa and neutrophils that governs neutrophil chemotaxis and apoptosis is disturbed by aberrant methylation of *PSD*, which may suppress the host immune system and result in UC-associated carcinogenesis.

## Figures and Tables

**Table I:** Clinicopathological characteristics of tumor specimens from UC patients with and without colorectal cancer.

	UCT	UCN	UCI
Number	N=6	N=6	N=15
Average age (years)	54.8±17.1	54.8±17.1	40.8±14.0
Gender			
Male	4	4	9
Female	2	2	6
Disease duration (years)	14.8±7.0	14.8±7.0	8.0±5.0^a^
*PSD* methylation (%)	71.4%	57.1%	27.3%

a Significantly short duration in UCN (= UCT) samples than UCI samples (P<0.05).

UCT, UC-associated colorectal cancer tissues; UCN, matched normal epithelia; UCI, non-neoplastic, UC epithelia.

**Table II:** Clinicopathological characteristics of tumor specimens from patients with UC-associated colorectal cancer.

Group	PSD	Age	Gender	Duration	Onset	Loc	Type	Dukes	INF	Ope
UCT1	M	77	M	13	64	R	Well	A	1	Total
UCT2	M	40	M	8	32	A	Muc	A	0	Total
UCT3	M	64	F	9	55	D	Well	A	1	Total
UCT4	U	35	M	15	20	D	Poor	D	0	Partial
UCT5	M	68	F	24	44	R	Well	B	0	Total
UCT6	U	45	M	25	20	D	Well	B	0	Total

UCT, UC-associated colorectal cancer tissues; UCN, matched normal epithelia; UCI, non-neoplastic UC epithelia; U in PSD, unmethylated; M in PSD, methylated; duration, disease duration (years); M in gender, male; F in gender, female; onset, age of onset (years); loc, location of carcinoma; A, D and R in loc, ascending colon, descending colon, and rectum, respectively; type, histological findings of carcinoma; well, poor, and muc in loc, well differentiated adenocarcinoma, poorly differentiated adenocarcinoma, and mucinous adenocarcinoma, respectively; Dukes, Dukes’ classification; INF, infiltration of neutrophils; ope, operation; total, partial, and right in ope, total colectomy, partial resection of the colon, and right hemi-colectomy, respectively.

## References

[1] Ekbom A, Helmick C, Zack M, Adami HO (1990). Ulcerative colitis and colorectal cancer. a population-based study. N Engl J Med.

[2] Lashner BA, Silverstein MD, Hanauer SB (1989). Hazard rates for dysplasia and cancer in ulcerative colitis. Results from a surveillance program. Dig Dis Sci.

[3] Duerr RH, Taylor KD, Brant SR (2006). A genome-wide association study identifies IL23R as an inflammatory bowel disease gene. Science.

[4] Franke A, Balschun T, Karlsen TH (2008). Replication of signals from recent studies of Crohn’s disease identifies previously unknown disease loci for ulcerative colitis. Nat Genet.

[5] Hugot JP, Chamaillard M, Zouali H (2001). Association of NOD2 leucine-rich repeat variants with susceptibility to Crohn’s disease. Nature.

[6] Barrett JC, Hansoul S, Nicolae DL (2008). Genome-wide association defines more than 30 distinct susceptibility loci for Crohn’s disease. Nat Genet.

[7] Hampe J, Franke A, Rosenstiel P (2007). A genome-wide association scan of nonsynonymous SNPs identifies a susceptibility variant for Crohn disease in ATG16L1. Nat Genet.

[8] Rioux JD, Xavier RJ, Taylor KD (2007). Genome-wide association study identifies new susceptibility loci for Crohn disease and implicates autophagy in disease pathogenesis. Nat Genet.

[9] Barrett JC, Lee JC, Lees CW (2009). Genome-wide association study of ulcerative colitis identifies three new susceptibility loci, including the HNF4A region. Nat Genet.

[10] Stoll M, Corneliussen B, Costello CM (2004). Genetic variation in DLG5 is associated with inflammatory bowel disease. Nat Genet.

[11] Humbert P, Russell S, Richardson H (2003). Dlg, Scribble and Lgl in cell polarity, cell proliferation and cancer. Bioessays.

[12] Wakabayashi M, Ito T, Mitsushima M (2003). Interaction of lp-dlg/KIAA0583, a membrane-associated guanylate kinase family protein, with vinexin and beta-catenin at sites of cell-cell contact. J Biol Chem.

[13] Okada S, Suzuki K, Kato T (2011). Aberrant methylation of the Pleckstrin and Sec7 domain-containing gene is implicated in ulcerative colitis-associated carcinogenesis through its inhibition of apoptosis. Int J Oncol.

[14] Eom YW, Yoo MH, Woo CH (2001). Implication of the small GTPase Rac1 in the apoptosis induced by UV in Rat-2 fibroblasts. Biochem Biophys Res Commun.

[15] Gulbins E, Coggeshall KM, Brenner B, Schlottmann K, Linderkamp O, Lang F (1996). Fas-induced apoptosis is mediated by activation of a Ras and Rac protein-regulated signaling pathway. J BiolChem.

[16] Esteve P, Embade N, Perona R (1998). Rho-regulated signals induce apoptosis in vitro and in vivo by a p53-independent, but Bcl2 dependent pathway. Oncogene.

[17] Servant G, Weiner OD, Herzmark P, Balla T, Sedat JW, Bourne HR (2000). Polarization of chemoattractant receptor signaling during neutrophil chemotaxis. Science.

[18] Servant G, Weiner OD, Neptune ER, Sedat JW, Bourne HR (1999). Dynamics of a chemoattractant receptor in living neutrophils during chemotaxis. Mol Biol Cell.

[19] Wang F, Herzmark P, Weiner OD, Srinivasan S, Servant G, Bourne HR (2002). Lipid products of PI(3)Ks maintain persistent cell polarity and directed motility in neutrophils. Nat Cell Biol.

[20] Rutter M, Saunders B, Wilkinson K (2004). Severity of inflammation is a risk factor for colorectal neoplasia in ulcerative colitis. Gastroenterology.

[21] Franco M, Peters PJ, Boretto J (1999). EFA6, a sec7 domain-containing exchange factor for ARF6, coordinates membrane recycling and actin cytoskeleton organization. EMBO J.

[22] Kurokawa K, Itoh RE, Yoshizaki H, Nakamura YO, Matsuda M (2004). Coactivation of Rac1 and Cdc42 at lamellipodia and membrane ruffles induced by epidermal growth factor. Mol Biol Cell.

[23] Boehm JE, Chaika OV, Lewis RE (1999). Rac-dependent anti-apoptotic signaling by the insulin receptor cytoplasmic domain. J BiolChem.

[24] Joneson T, Bar-Sagi D (1999). Suppression of Ras-induced apoptosis by the RacGTPase. Mol Cell Biol.

[25] Nishida K, Kaziro Y, Satoh T (1999). Anti-apoptotic function of Rac in hematopoietic cells. Oncogene.

[26] Saez R, Chan AM, Miki T, Aaronson SA (1994). Oncogenic activation of human R-ras by point mutations analogous to those of prototype H-ras oncogenes. Oncogene.

[27] Wang HG, Millan JA, Cox AD (1995). R-Ras promotes apoptosis caused by growth factor deprivation via a Bcl-2 suppressible mechanism. J Cell Biol.

[28] Srinivasan S, Wang F, Glavas S (2003). Rac and Cdc42 play distinct roles in regulating PI(3,4,5)P3 and polarity during neutrophil chemotaxis. J Cell Biol.

[29] Lampinen M, Ronnblom A, Amin K (2005). Eosinophil granulocytes are activated during the remission phase of ulcerative colitis. Gut.

[30] Glogauer M, Marchal CC, Zhu F (2003). Rac1 deletion in mouse neutrophils has selective effects on neutrophil functions. J Immunol.

[31] Roessner A, Kuester D, Malfertheiner P, Schneider-Stock R (2008). Oxidative stress in ulcerative colitis-associated carcinogenesis. Pathol Res Pract.

